# Calorie Restriction-Mediated Replicative Lifespan Extension in Yeast Is Non-Cell Autonomous

**DOI:** 10.1371/journal.pbio.1002048

**Published:** 2015-01-29

**Authors:** Szu-Chieh Mei, Charles Brenner

**Affiliations:** 1 Department of Biochemistry, Carver College of Medicine, University of Iowa, Iowa City, Iowa, United States of America; New York University, UNITED STATES

## Abstract

In laboratory yeast strains with Sir2 and Fob1 function, wild-type NAD^+^ salvage is required for calorie restriction (CR) to extend replicative lifespan. CR does not significantly alter steady state levels of intracellular NAD^+^ metabolites. However, levels of Sir2 and Pnc1, two enzymes that sequentially convert NAD^+^ to nicotinic acid (NA), are up-regulated during CR. To test whether factors such as NA might be exported by glucose-restricted mother cells to survive later generations, we developed a replicative longevity paradigm in which mother cells are moved after 15 generations on defined media. The experiment reveals that CR mother cells lose the longevity benefit of CR when evacuated from their local environment to fresh CR media. Addition of NA or nicotinamide riboside (NR) allows a moved mother to maintain replicative longevity despite the move. Moreover, conditioned medium from CR-treated cells transmits the longevity benefit of CR to moved mother cells. Evidence suggests the existence of a longevity factor that is dialyzable but is neither NA nor NR, and indicates that Sir2 is not required for the longevity factor to be produced or to act. Data indicate that the benefit of glucose-restriction is transmitted from cell to cell in budding yeast, suggesting that glucose restriction may benefit neighboring cells and not only an individual cell.

## Abstract 2

Calorie-restriction extends lifespan in many multicellular organisms; here substances secreted by calorie-restricted yeast are found to induce longer life in other yeast cells, suggesting that cellular communication is a component of this phenomenon even in a single-celled organism.

## Author Summary

Though calorie restriction extends lifespan and healthspan in multiple model organisms, the intrinsic mechanisms remain unclear. In budding yeast *Saccharomyces cerevisiae*, manipulation of nicotinamide adenine dinucleotide (NAD^+^)—a central metabolic cofactor—can restrict or extend replicative lifespan, suggesting that NAD^+^-dependent targets might be mediators of extended longevity. However, although treating cells with the NAD^+^ precursor nicotinamide riboside extends lifespan, intracellular NAD^+^ metabolites levels are not altered by glucose restriction. This suggests the potential involvement of extracellular factors in replicative lifespan extension. Here we show that though yeast cells display a longevity benefit upon glucose restriction, these cells surprisingly lose the longevity benefit if moved from their local environment to fresh glucose-restricted media. They are, however, able to regain the longevity benefit, despite the change in environment, if the new environment is supplemented with conditioned medium from glucose restricted cells. Our results suggest that calorie restriction-induced longevity is not cell autonomous and, instead, appears to be transmitted from cell to cell in *S. cerevisiae* via a dialyzable extracellular factor.

## Introduction

Calorie restriction (CR) extends lifespan and healthspan in several model organisms [1, 2]. Although inconsistent results have been obtained on lifespan extension in primates, the beneficial effects on healthspan are widely observed [3, 4]. Thus, dissecting the underlying mechanism of how CR contributes to health is of substantial interest.

Two different lifespan paradigms are commonly employed in the yeast *Saccharomyces cerevisiae*. Replicative lifespan (RLS), a model for understanding aging of dividing cells, is defined as the number of divisions that a yeast mother cell undergoes prior to senescence [5, 6]. Chronological lifespan, considered to be more relevant to post-mitotic cells, measures the duration of cell viability during stationary phase [7].

In yeast, CR is achieved by reducing glucose concentration from 2% to 0.5% or below [8]. The *SIR2* gene, which encodes an NAD^+^-dependent protein lysine deacetylase, and functional NAD^+^ salvage genes were shown to be required for CR-mediated RLS extension in strain backgrounds containing a wild-type *FOB1* gene [8–10]. Moreover, addition of nicotinamide riboside (NR) extends yeast lifespan concomitant with increased Sir2-dependent functions and elevated intracellular NAD^+^[11]. CR-mediated changes in NAD^+^ metabolites were proposed to exist and underlie the longevity benefit of CR [10, 12]. However, sensitive and quantitative liquid chromatography mass-spectrometry (LC-MS) methods [13, 14] have been developed to measure the NAD^+^ metabolome during normal and CR conditions [13]. Though all NAD^+^ metabolites were increased by addition of nicotinic acid (NA)—a condition that extends lifespan—the levels of intracellular NAD^+^ metabolites did not change upon CR [13]. Careful experiments have established that Sir2 and CR work in parallel pathways [15] and that exogenously added nicotinamide (Nam), initially proposed to function as a Sir2 inhibitor in high glucose [10], blocks the longevity benefit of CR in yeast strains without Sir2 [16].

To dissect the complexities of CR, we developed methods to quantify the enzymes that participate in the reactions of the NAD^+^ metabolome and discovered that Sir2 and Pnc1, which successively convert NAD^+^ to Nam and NA, are up-regulated during CR [17]. If the effects of glucose restriction on NAD^+^ metabolism were to promote conversion of NAD^+^ to NA, one might expect to see a change in levels of intracellular NAD^+^ metabolites, such as NA. However, existing NAD^+^ metabolome data are inconsistent with such intracellular changes [13]. Previous experiments indicated that when yeast cells are provided with extracellular Nam, it is imported, converted intracellularly to NA by Pnc1, and then exported to the culture media in a manner that maintains intracellular NAD^+^ homeostasis [18]. We therefore questioned whether glucose restriction might result in increased net conversion of NAD^+^ to NA accompanied by export of NA. If this were the case, then young mother cells might export NA in order to use it later in life.

In this study, we aimed to test the hypothesis that glucose-restricted mother cells export metabolites, such as NA, for replication of older cells. By performing a modified RLS assay in which aged mother cells were moved away from their original locations to new locations on the same plate, we discovered that moving mother cells diminished the longevity benefit of glucose-restriction. However, supplementation with NA, NR, or conditioned medium from glucose-restricted cells restores the longevity benefit. This longevity-promoting activity is dialyzable and does not require Sir2 for production or action.

Taken together, our data suggest that glucose restriction benefits entire colonies rather than single, glucose-restricted cells. Though a yeast colony is clonal and largely contains cells of identical genotype, this mechanism seems to bear some features of altruism [19–21].

## Results

### Glucose-Restricted and CR-Mimetic Mother Cells Lose the Longevity Benefit of CR with Migration to Fresh Plate Locations

**Figure 1:**
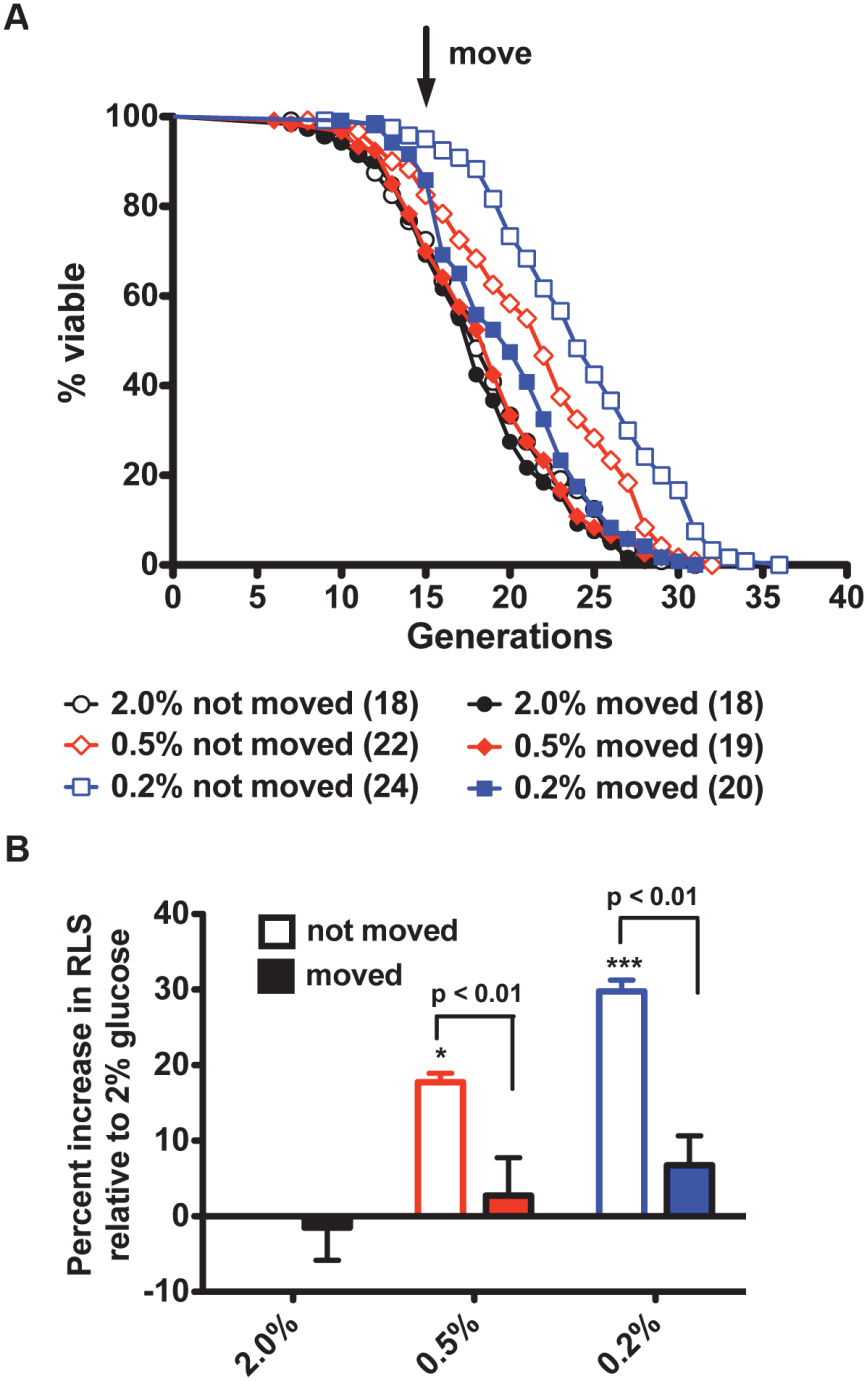
Moving glucose-restricted mother cells to new plate locations largely negates the longevity benefit of CR. (A) RLS analysis for wild-type strain BY4741 in 2% glucose and glucose-restricted conditions indicates that the longevity benefit of CR is lost upon migration to new plate locations. (B) Percent increases in median life span relative to RLS on 2% glucose without migration. Data were collected from four independent experiments with *n* = 30. *, *p* < 0.05; ***, *p* < 0.001 in comparison to the 2% not moved group.

To test the hypothesis that conditioned medium from glucose-restricted cells is essential for CR-mediated lifespan extension, we modified the RLS paradigm in a manner that permits evaluation of conditioned medium. Yeast mother cells were arrayed on 2%, 0.5%, or 0.2% glucose-containing yeast extract/peptone/dextrose (YPD) plates and were subjected to the typical routine of daughter cell removal in every generation. When most mother cells had produced 15 generations of daughters, half of the aged mother cells were moved to fresh locations on the same plate—to avoid bias in moving mother cells, the mothers to be moved were chosen prior to aging. As shown in Fig. 1, yeast mother cells cultured on 2% glucose YPD plates were unaffected by migration to new plate locations. In contrast, cells that were restricted to 0.5% or 0.2% glucose obtained a 20%–30% increase in lifespan with respect to 2% glucose-grown cells only if they remained in their original plate locations. Mother cells lost the longevity benefit of CR if they were moved to new plate locations with the same restricted concentrations of glucose.

Replicative longevity is exhibited by yeast strains that are either genetically deleted for glucose and growth control pathways, termed CR-mimetic strains, or by longevity pathways that run in parallel to CR [15]. If moving mother cells diminishes longevity owing to loss of a CR-induced factor, then CR-mimetic strains should show extended lifespan on 2% glucose plates that is diminished by moving mother cells. As shown in S1A Fig., the control strain BY4742 is unaffected by moving when cultured on 2% glucose YPD plate. However, this strain exhibited a longevity benefit on 0.2% glucose media that was lost upon migration. Moreover, as shown in S1B Fig., deletion of *sch9* [22], *tor1* [22], or *hxk2* [15] extended lifespan on 2% glucose in a manner that was diminished by 67% upon moving. As shown in S1C Fig., consistent with the idea that deletion of *fob1* and overexpression of *SIR2* produce extensions in RLS that are not related to CR [15], these strains exhibited 30% and 20% extended lifespan compared to the BY4742 control strain on 2% glucose whether mothers were moved or allowed to remain on their original plate locations.

These results indicate that wild-type yeast mother cells on 2% glucose media and strains with CR-unrelated longevity pathways are unaffected by moving while glucose-restricted and CR-mimetic mother cells lose a longevity benefit upon moving. These data suggest that CR mother cells are either hypersensitive to physical movement or rely on a component of conditioned media for longevity.

### NA and NR Supplementation Restore Lifespan Extension to CR Mother Cells

**Figure 2:**
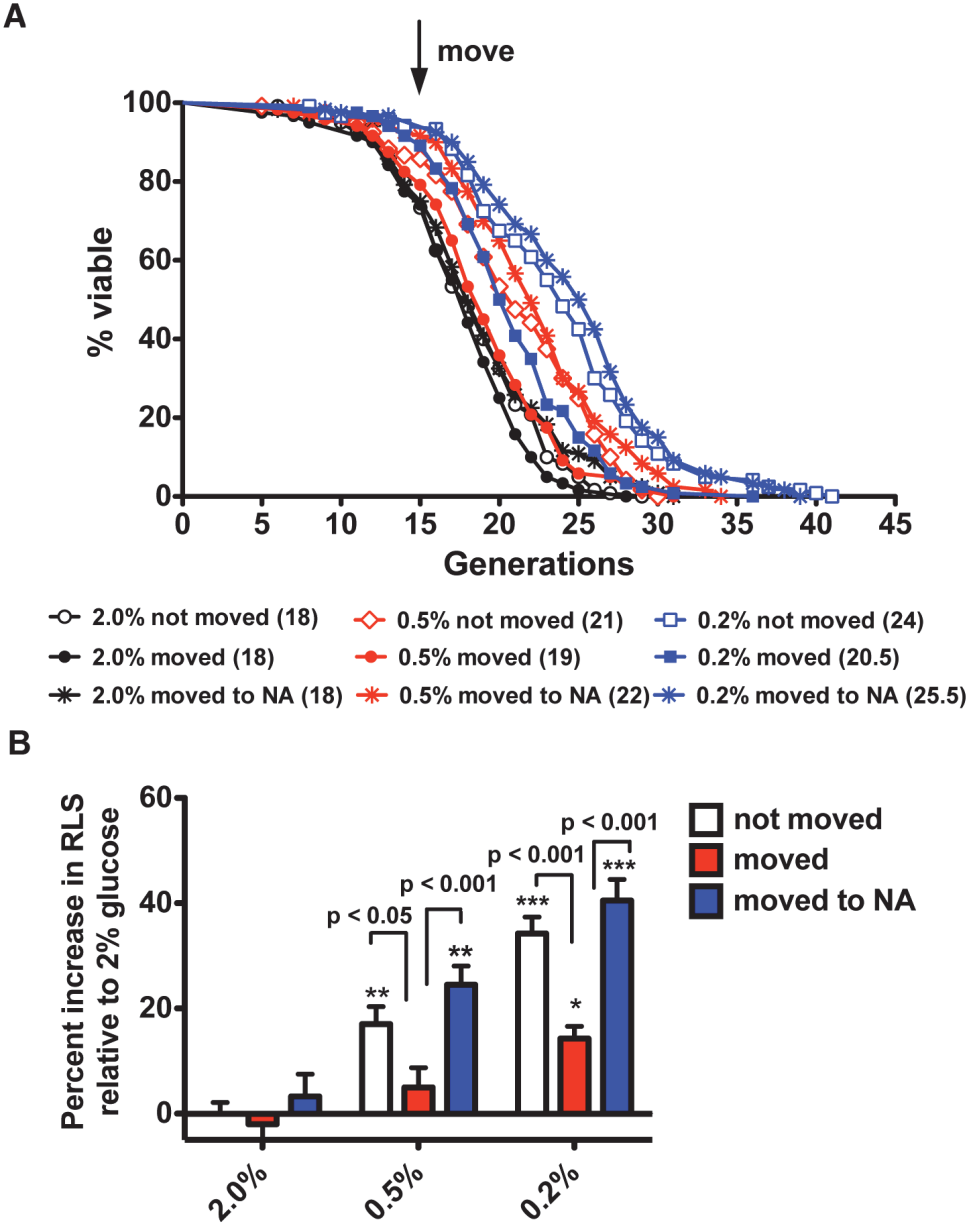
Supplementation with NA restores the longevity benefit to moved glucose-restricted cells. (A) RLS analysis for wild-type BY4741 in 2% glucose and glucose-restricted conditions indicates that supplementation with NA is sufficient to restore the longevity benefit of CR upon migration to new plate locations. (B) Percent increases in median life span relative to RLS on 2% glucose without migration. Data were collected from four independent experiments with *n* = 30. *, *p* < 0.05; **, *p* < 0.01; *** p, < 0.001 in comparison to the 2% not moved group.

Because mother cells on 2% glucose media did not have their lifespan degraded by migration to fresh plate locations, it seemed unlikely that yeast mother cells are hypersensitive to migration. Instead, we suspected that a longevity-promoting substance was left behind in conditioned media. Since the enzymes to convert NAD^+^ to NA were up-regulated in CR mother cells [17] and NA supplementation to aged mother cells could function to elevate NAD^+^ synthesis and Sir2 activity, as precedented by the effects of NR on yeast cell RLS in high glucose [11], we aimed to test NA as a candidate longevity factor. To test whether NA could complement the loss of RLS in migrated CR mother cells, we performed an RLS experiment at three concentrations of glucose in which one-third of the mother cells at 15 generations were moved to fresh locations on the same plate, and one-third of the mother cells were migrated to fresh locations of media containing 0.5 mM NA—as in all similar experiments, the mothers to be moved were preselected in order to avoid experimental bias. The data indicate that mother cells grown in 0.5% and 0.2% glucose consistently lose the longevity benefit of CR. However, supplementation with NA was sufficient to maintain the longevity benefit of CR despite a move (Fig. 2). Because NR metabolism was reported to be essential for CR-mediated lifespan extension [23], we also tested whether NR could provide the same longevity benefit. As shown in S2 Fig., NR supplementation provided similar rescue activity as NA. These data defeat the idea that CR mother cells are hypersensitive to migration. Moreover, these data indicate that extracellular supplementation of NA or NR is capable of restoring lifespan extension to migrated CR mother cells. Interestingly, addition of NR extends RLS of yeast mother cells grown in 2% glucose when provided at initiation of aging [11]. However, as shown in S2 Fig. and Fig. 2, when mother cells were aged for 15 generations on 2% glucose, addition of NA or NR to fresh plate locations was incapable of rejuvenating these cells.

### Calorie Restricted Conditioned Media Restore the Longevity Benefit of CR

Since glucose restriction up-regulated expression of enzymes that convert NAD^+^ to NA [17] and NA addition maintained RLS, NA emerged as an obvious candidate transmissible factor, which might mediate the longevity benefit of CR. To address this question, we prepared 30 ml cultures of yeast at final OD_600 nm_ of 0.5 in which the glucose concentrations were 2%, 0.5%, and 0.2%. The three conditioned media samples plus a 30 ml sample of YP without glucose in which yeast cells were not grown were analyzed by LC-MS as described [14]. As shown in S3 Fig., the NA concentration in YP media and in each of the three conditioned media samples was between 37 µM and 43 µM. NR concentrations were below the detection limit (<0.04 µM) in all conditions examined. This experiment does not provide experimental support for NA as a molecule that is exported by glucose-restricted cells. However, it is possible that isolated glucose-restricted cells export NA and that the effect is masked by simultaneous vitamin import and export in a flask-grown culture, which contains a mixture of aged and young mother cells.

**Figure 3:**
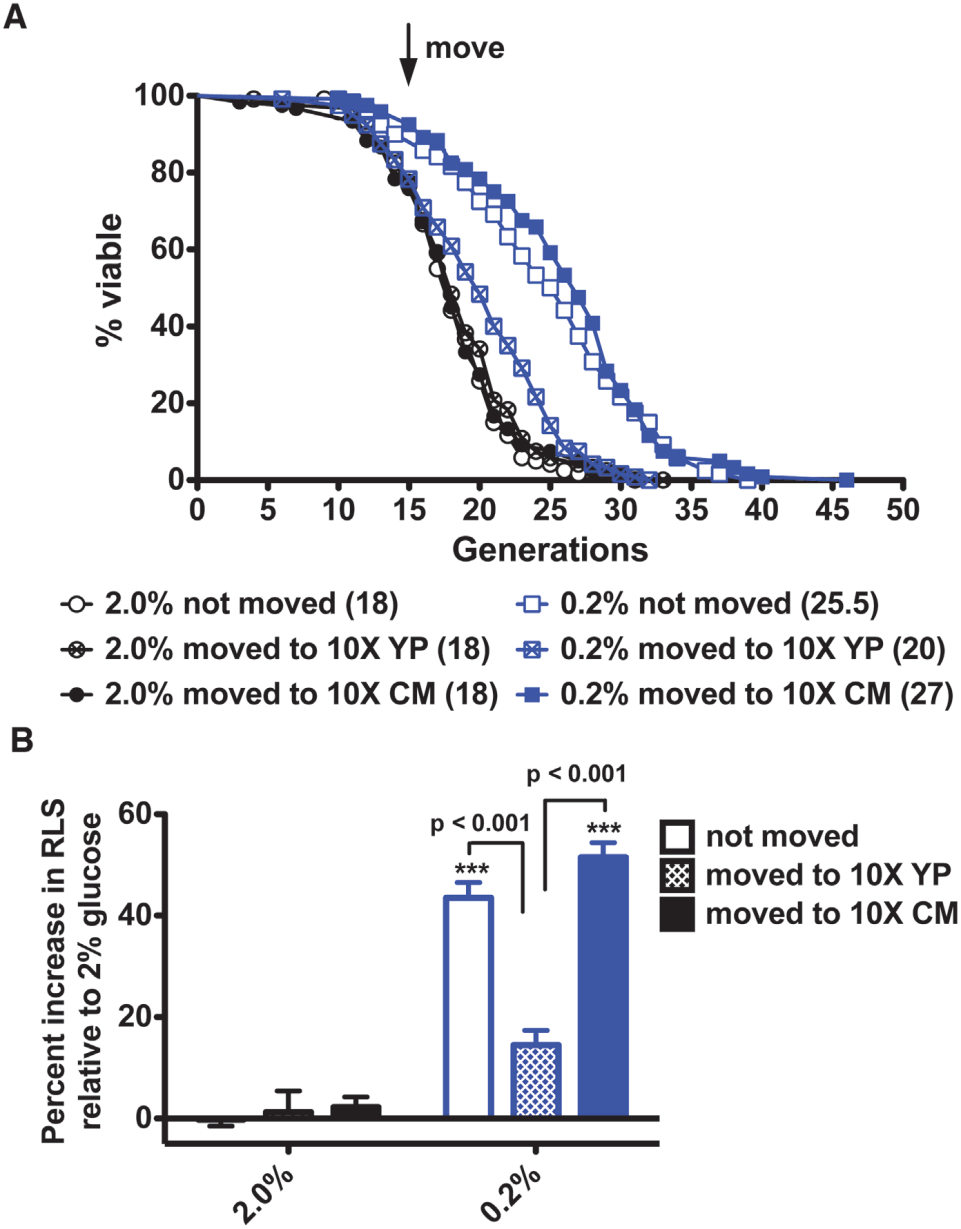
Supplementation with concentrated conditioned media restores the longevity benefit to moved glucose-restricted cells. (A) RLS analysis for wild-type BY4741 in 2% glucose and glucose-restricted conditions indicates that supplementation with concentrated conditioned media is sufficient to restore the longevity benefit of CR upon migration to new plate locations. (B) Percent increases in median life span relative to RLS on 2% glucose without migration. Data were collected from four independent experiments with *n* = 30. ***, *p* < 0.001 in comparison to the 2% not moved group.

To test whether a flask-grown culture of glucose-restricted cells contains a factor that allows lifespan to be extended despite migration, we inoculated wild-type yeast in 2% or 0.2% glucose-containing YPD media, allowed cells to grow until the glucose was undetectable and then collected and lyophilized the media. To control for the RLS effects of salt or other medium components, nonconditioned YP media without glucose were also lyophilized as a control. Lyophilized media, reconstituted in water, were applied to sections of 2% and 0.2% glucose-containing YP plates on which the modified RLS assay was performed. As shown in Fig. 3, when mother cells began their RLS on 2% glucose media, they had a short lifespan whether they remained in place, were moved to nonconditioned media, or were moved to media from 2% glucose-grown yeast. When mother cells began their RLS on 0.2% glucose media and were moved to nonconditioned media, they lost 20% of their lifespan, just as though they had been moved to new plate locations without supplementation. However, when glucose-restricted mother cells were moved to new plate locations supplemented with concentrated media from glucose-restricted cells, lifespan was maintained and, in fact, extended by about 10%. These data indicate that glucose restriction may result in production of a transmissible factor required for extension of lifespan.

### A Small Molecule Promotes RLS Extension

Since CR-conditioned media could restore and provide an increased longevity benefit to migrated CR mother cells, we wished to distinguish between a longevity factor in CR-conditioned media and the absence of a harmful factor in 2% glucose-conditioned media. We therefore moved yeast mother cells every generation on both 2% and 0.2% glucose plates so that yeast mother cells were always maintained in fresh environments. As shown in S4 Fig., lifespan for yeast mother cells grown on 2% glucose was not changed whether kept in the same locations or always-fresh environments. This result eliminated the possibility of a diffusible harmful factor produced by non-moved mother cells in high glucose. In contrast, the lifespan of yeast mother cells on 0.2% glucose dropped significantly compared to non-moved mother cells (S4 Fig.). Because CR mother cells moved at 15 generations retained a 7% benefit (Fig. 1) while CR mother cells moved in every generation obtained no benefit, it appears that prolonged exposure to a longevity factor helps CR mother cells survive old age.

**Figure 4:**
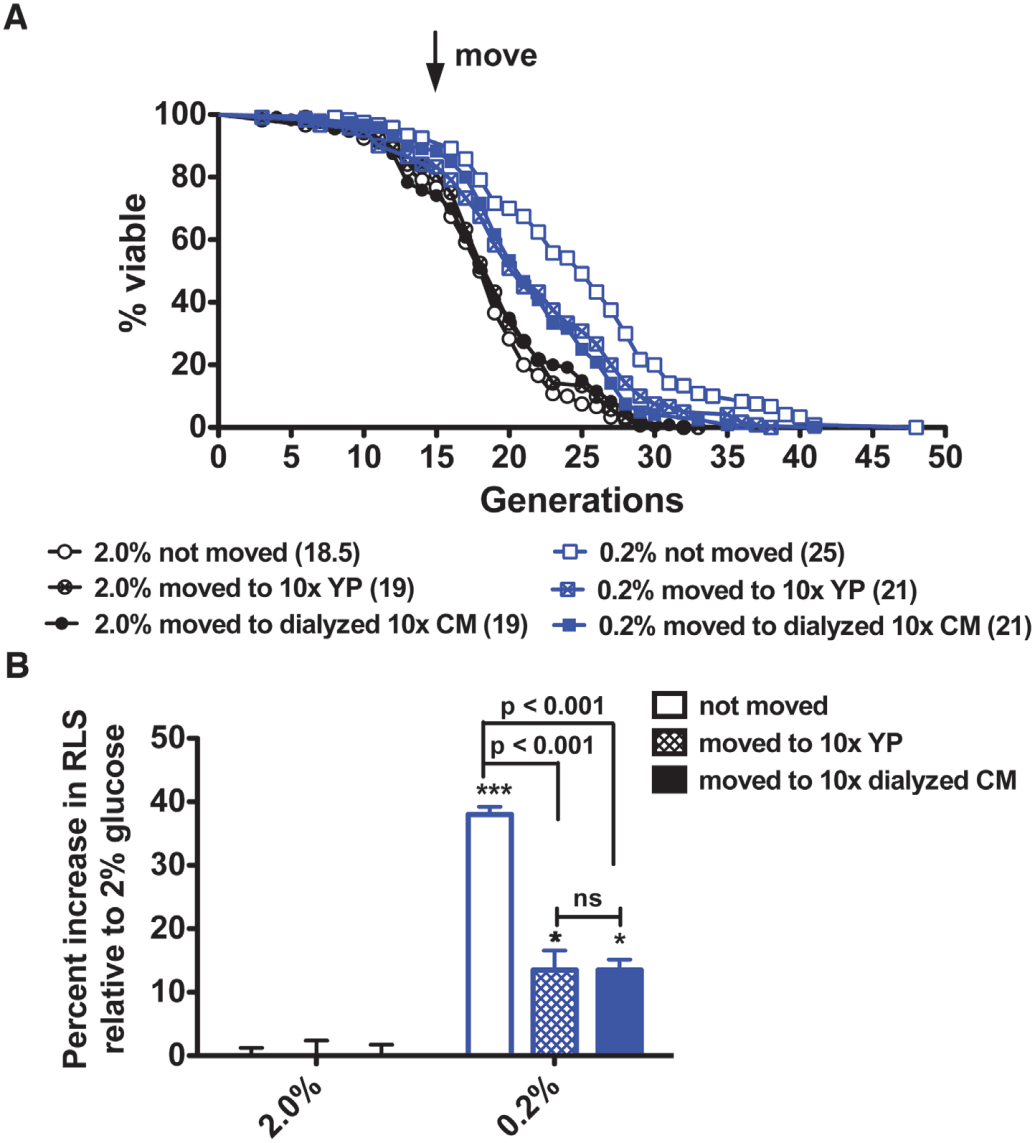
A low molecular weight factor in CR-conditioned media is required for the longevity benefit of CR. (A) RLS analysis for wild-type BY4741 in dialyzed 2% glucose and glucose-restricted conditions indicates that the longevity factor is a small molecule. (B) Percent increases in median life span relative to RLS on 2% glucose without migration. Data were collected from four independent experiments with *n* = 30. ns, no significant difference; *, *p* < 0.05; ***, *p* < 0.001 in comparison to the 2% not moved group.

We therefore collected conditioned media and nonconditioned YP media without glucose and removed small molecules with 3.5 kDa cutoff dialysis cassettes. After dialysis, media samples were lyophilized, suspended in water, and applied to plates. As shown in Fig. 4, dialyzed conditioned media from 2% glucose or 0.2% glucose-grown cells were inactive at altering RLS. Because dialyzed conditioned media from high glucose-grown yeast did not gain a lifespan extending activity upon dialysis, there is no evidence of a harmful substance in the 2% glucose conditioned media. However, loss of RLS-extending activity in low glucose-grown culture media subjected to dialysis suggests a low molecular weight transmissible factor.

### Sir2 Is Dispensable for CR Mother Cells to Produce the Longevity Factor

**Figure 5:**
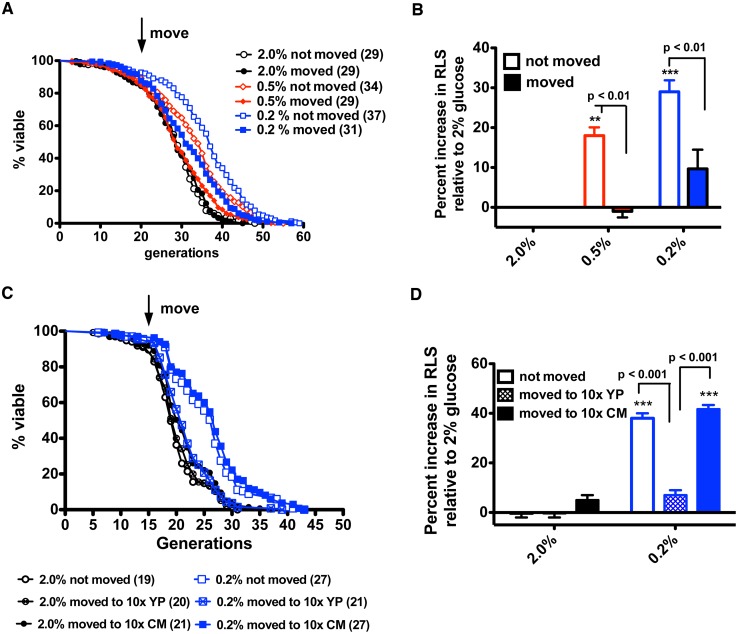
Sir2 is not required for production or action of the longevity factor. (A) RLS analysis for *sir2 fob1* double mutant yeast in 2% glucose and glucose-restricted conditions indicates that the longevity benefit of CR is lost upon migration to new plate locations without Sir2. (B) Percent increases in median life span relative to RLS on 2% glucose without migration. Data were collected from three independent experiments with *n* = 60. **, *p* < 0.01; *** *p* < 0.001 in comparison to the 2% not moved group. (C) RLS analysis for wild-type BY4741 in 2% glucose and glucose-restricted conditions indicates that supplementation with concentrated conditioned media from *sir2 fob1* mutant yeast restores longevity benefit of CR upon migration to new plate locations. (D) Percent increases in median life span relative to RLS on 2% glucose without migration. Data were collected from three independent experiments with *n* = 45. ***, *p* < 0.001 in comparison to the 2% not moved group.

Sir2 activity was initially proposed to be crucial for CR-mediated lifespan extension in yeast [8]. However, Sir2 is not required for CR-mediated lifespan extension in strains lacking *FOB1* [15]. Because the observation that CR-induces up-regulation of Sir2 and Pnc1 [17] led to the idea that young mother cells might export a longevity factor, we tested whether Sir2 is required for this process. Consistent with previous reports [15], we observed a 20%–30% lifespan extension for the *sir2 fob1* double mutant yeast strain (KK144) on 0.5% or 0.2% glucose compared to lifespan on 2% glucose (Fig. 5A and 5B). However, moving the double mutant yeast strain to fresh locations completely negated the CR benefit (Fig. 5A and 5B). Further, we prepared conditioned media from the *sir2 fob1* double mutant yeast strain grown in 2% or 0.2% glucose. The conditioned media were then applied to sections of 2% and 0.2% glucose-containing YP plates on which the modified RLS assay was performed. As shown in Fig. 5C and 5D, glucose-restricted wild-type mother cells moved to plate locations supplemented with conditioned media from glucose-restricted *sir2 fob1* double mutant yeast showed similar lifespans as wild-type cells that were not moved. Because glucose-restricted conditioned media from *sir2* mutant yeast have longevity factor activity, Sir2 is dispensable for producing, exporting, and utilizing this activity.

## Discussion

The underlying mechanism of how CR extends lifespan in yeast remains incompletely understood despite mutational data in support of requirements for functional NAD^+^ salvage [8–10]. Multiple studies have shown that levels of NAD^+^ and/or levels of the collection of intracellular NAD^+^ metabolites are not greatly altered by CR in flask-grown cultures of yeast [9, 10, 12, 13, 24]. However, Sir2 and Pnc1, which successively convert NAD^+^ to Nam and NA, are increased in protein expression in CR [17]. Because NA does not increase intracellularly during CR [13], we considered whether NA or another compound might be exported by a young mother cell and taken up later in order to survive old age. Our data indicate that media from glucose-restricted mother cells are necessary (Fig. 1) and sufficient (Fig. 3) for CR-induced lifespan extension and this requirement is independent of Sir2 to produce or respond to the activity (Fig. 5). Moreover, we showed that moving to new plate locations only affect CR-mediated lifespan extension while showing no effect on CR-unrelated long-lifespan strains (S1 Fig.). Interestingly, microfluidic technologies have recently been applied to characterize yeast RLS. Whereas, the lifespan extension exhibited by *fob1* deletion is readily apparent in microfluidic assays [25, 26], the extension of lifespan due to CR disappeared [27]. Though the latter microfluidic assays led to questions about whether CR-induced lifespan extension is real [27], microfluidic dissection does not, by its nature, maintain the micro-environment of CR mother cells because media are continuously streamed away, much like our experiment in which mother cells are moved in every generation (S4 Fig.). This result provides additional literature support for the existence of a transmissible longevity factor.

CR has been shown to extend yeast lifespan by regulating vacuolar acidity [28]. If compartmental pH differs as a function of glucose, it is possible that extracellular pH value is also altered. However, a recent study has shown that buffering the pH of culture media does not extend yeast RLS [29]. This suggests that CR-induced RLS transmission is not simply a function of pH.

Like conditioned media from glucose-restricted cells, provision of NA or NR allowed yeast mother cells to be migrated from their original plate locations in a manner that preserved an increase in lifespan (S1 Fig. and Fig. 2). Export of NA has been previously observed [18] and extension of lifespan with an NAD^+^ precursor vitamin is precedented by the effect of NR [11]. However, we could not detect a significant change in the levels of these vitamins in flask-grown cells. Because acetylation of telomeric histone H4 in yeast has been shown to correlate with aging [30], it is possible that the transmissible activity directly or indirectly inhibits this process. This could account for why NA and NR have a mimetic activity though the endogenous mechanism would not require Sir2.

Cell to cell transmission of the beneficial effect of CR was not anticipated. Though the cells in a colony are clonal, the activity suggests characteristics that could be termed altruistic, especially if there are costs to transmit the activity. Alternatively, the activity may simply spill over from cell to cell and produce a community benefit without substantial cellular costs. In a cell-autonomous context, the ability of damaged yeast cells to undergo programmed cell death has been termed altruistic because it produces surviving cells with reduced damage [21, 31]. Chemical characterization of the transmissible mediator of CR-promoted lifespan extension is now a major focus area.

## Materials and Methods

### Strains and Media

Yeast strains used in this study are listed in Table 1. Medium used for RLS analysis was YP (2% bacto peptone, 1% yeast extract) supplemented with filter-sterilized glucose at final concentrations of 2%, 0.5%, or 0.2%.

**Table 1:** Yeast strains used in this study.

**Strain**	**Genotype**
BY4741	MAT**a** *his3*Δ1 *leu*2Δ0 *lys*2Δ0 *ura*3Δ0
BY4742	MAT**α** *his*3Δ1 *leu*2Δ0 *lys*2Δ0 *ura*3Δ0
DC:110G4	BY4742 *fob1*Δ::*KanMX*
KK229	BY4742 *SIR2/LEU2*
KS68	BY4742 *sch9*Δ::*HIS3*
DH461	BY4742 *tor1*Δ::*URA3*
DC:122H10	BY4742 *hxk2*Δ::*KanMX*
KK144	BY4742 *sir2*Δ::HIS3 *fob1*Δ::LEU2

### Preparation of Conditioned Media

Single colonies of wild-type or *sir2 fob1* yeast strains were inoculated in 5 ml 2% glucose-containing YPD media and allowed to grow until OD_600 nm_ reached 0.5. Cells were then inoculated into 50 ml 2% or 0.2% glucose-containing YPD media at an initial OD_600 nm_ of 0.01 and were grown until glucose was undetectable by Glucose Colorimetric Assay kit (Cayman Chemical). After centrifugation, culture supernatants were transferred to new tubes. Some samples were dialyzed twice with 3,500 MWCO Slide-A-Lyzer Dialysis cassettes (Thermo Scientific) against 1 liter non-conditioned YP media without glucose at 4°C. All conditioned media were lyophilized and resuspended in water at 1/10 volume of the original media samples. Note that when conditioned medium is provided to yeast mother cells in a RLS analysis, conditioned medium is always derived from a culture grown at the same concentration of glucose.

### RLS Analysis

Experiments were carried out as described [5] with some modification (S5 Fig.). To blind the experiments, the plates and/or supplementations were prepared by other laboratory members and coded. In brief, 60 cells were arrayed on one part of a YP plate with 2%, 0.5%, or 0.2% glucose. Prior to any cell division, 30 mother cells were assigned the group that would be migrated when these cells reach 15 generations (20 generations for BY4742 background cells). Such mother cells were moved to new locations on the same plate. For NA-supplementation experiments, 90 mother cells were arrayed on one part of the YP plate with 2%, 0.5%, or 0.2% glucose. After scoring the plate into three sectors, 30 mother cells were assigned to groups that would not be moved, moved to new locations on the same plate on a sector to which 200 µl of water was applied, or moved to new locations on a sector to which 200 μl of 0.5 mM NA had been applied. Migrations were effected after mother cells reached 15 generations. Conditioned media experiments were performed in a similar manner. Ninety mother cells were arrayed on plates with either 2% or 0.2% glucose. Thirty mother cells were moved to a sector to which 200 μl of 10×-concentrated non-conditioned YP had been applied. Thirty mother cells were moved to a sector to which 200 μl of 10×-concentrated conditioned media (10× CM) from cells grown in the same glucose concentration was applied. In all cases, the author performing RLS was blinded with respect to plate conditions. Survival curves were plotted with all data collected from four independent experiments (120 mothers in total), and RLS data were plotted as percent increases in median RLS compared to non-moved mother cells aging in 2% glucose media. Data are means and standard deviations from independent RLS experiments and statistical data were analyzed by one-way ANOVA. Raw data for all RLS experiments are available in S1 Data.

## Supporting Information

S1 Data Raw data for RLS experiments. (TXT)

S1 Fig Moving CR-mimetic mother cells to new plate locations negates the longevity benefit of *sch9*, *tor1*, and *hxk2* deletions. (A) RLS analysis for wild-type strain BY4742 in 2% and 0.2% glucose indicates that the longevity benefit of CR is lost upon migration to new plate locations. (B) RLS analysis for BY4742 and three CR-mimetic strains indicates that the longevity benefit is lost upon migration to new plate locations in 2% glucose media. (C) RLS analysis of *fob1* and *SIR2*-overexpressing strains in the BY4742 background indicates that long-lived yeast strains with genetic alterations termed CR-unrelated [15] are unaffected by moving mothers on 2% glucose. *n* = 45 for each condition of each strain.
(TIF)

S2 Fig Supplementation with NR restores the longevity benefit to moved glucose-restricted cells. RLS analysis for wild-type BY4741 in 2% glucose and glucose-restricted conditions indicates that supplementation with NR is sufficient to restore the longevity benefit of CR upon migration to new plate locations. *n* = 45 for each condition.
(TIF)

S3 Fig NA concentrations in conditioned media. BY4741 and KK144 yeast strains were cultured from OD_600 nm_ 0.0005 to 0.5 in YP media with 2%, 0.5%, or 0.2% glucose. NA concentration from these media and nonconditioned YP were determined by LC-MS [13] with pure NA as standard.
(TIF)

S4 Fig A constantly changed environment does not rejuvenate high glucose-grown mother cells. RLS analysis for wild-type BY4741 in 2% glucose and glucose-restricted conditions indicates that keeping yeast mother cells in an always-fresh environment by moving at each generation does not extend lifespan of cells grown at 2% glucose and negates the longevity benefit to glucose-restricted cells. *n* = 45 for each condition.
(TIF)

S5 Fig Diagram of modified RLS assay. (A) New yeast mother cells were grouped and assigned to be moved or not at the beginning of each RLS experiment. After reaching 15 generations, mother cells assigned to be moved were moved to new locations on the same plate. (B) New yeast mother cells were arrayed on a sector of a plate without supplementation, grouped and assigned to be moved to sectors on the same plate with indicated supplementation or not to be moved. Mother cells to be moved were moved after 15 generations. Water was a control for NA and NR. 10× YP was a control for non-dialyzed or dialyzed conditioned media samples.
(TIF)

## Abbreviations

CR: calorie restriction
LC-MS: liquid chromatography mass-spectrometry
NA: nicotinic acid
Nam: nicotinamide
NR: nicotinamide riboside
RLS: replicative lifespan
YPD: yeast extract/peptone/dextrose
