# Supplementary figures and images for: Calorie Restriction-Mediated Replicative Lifespan Extension in Yeast Is Non-Cell Autonomous
Source: PLoS Biol. 2015 Jan 29;13(1):e1002048. doi: 10.1371/journal.pbio.1002048 (PMC4310591; doi:10.1371/journal.pbio.1002048)

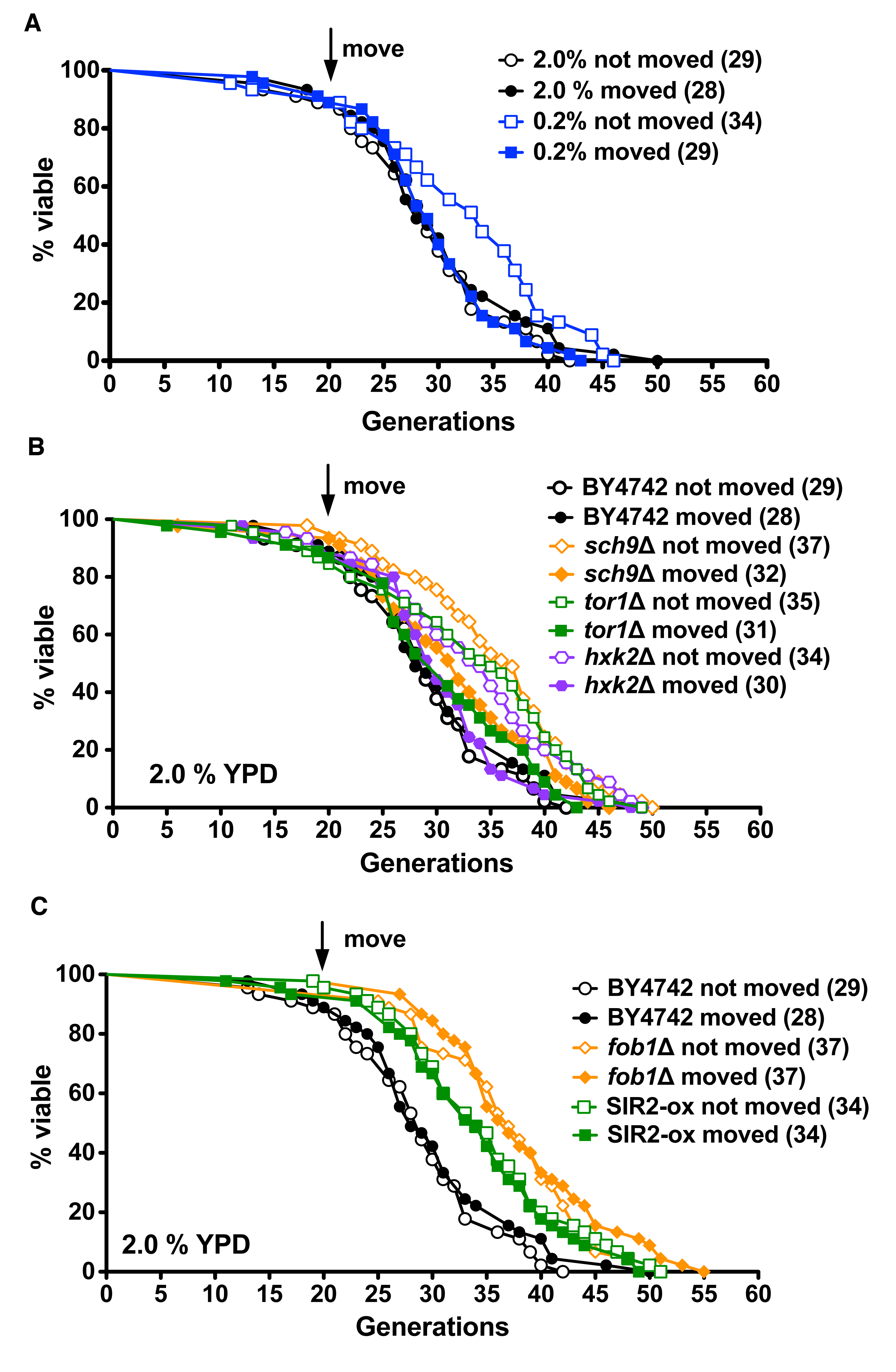

Supplement: S1 Fig — (A) RLS analysis for wild-type strain BY4742 in 2% and 0.2% glucose indicates that the longevity benefit of CR is lost upon migration to new plate locations. (B) RLS analysis for BY4742 and three CR-mimetic strains indicates that the longevity benefit is lost upon migration to new plate locations in 2% glucose media. (C) RLS analysis of fob1 and SIR2-overexpressing strains in the BY4742 background indicates that long-lived yeast strains with genetic alterations termed CR-unrelated [15] are unaffected by moving mothers on 2% glucose. n = 45 for each condition of each strain. (TIF) [file pbio.1002048.s002.tif]

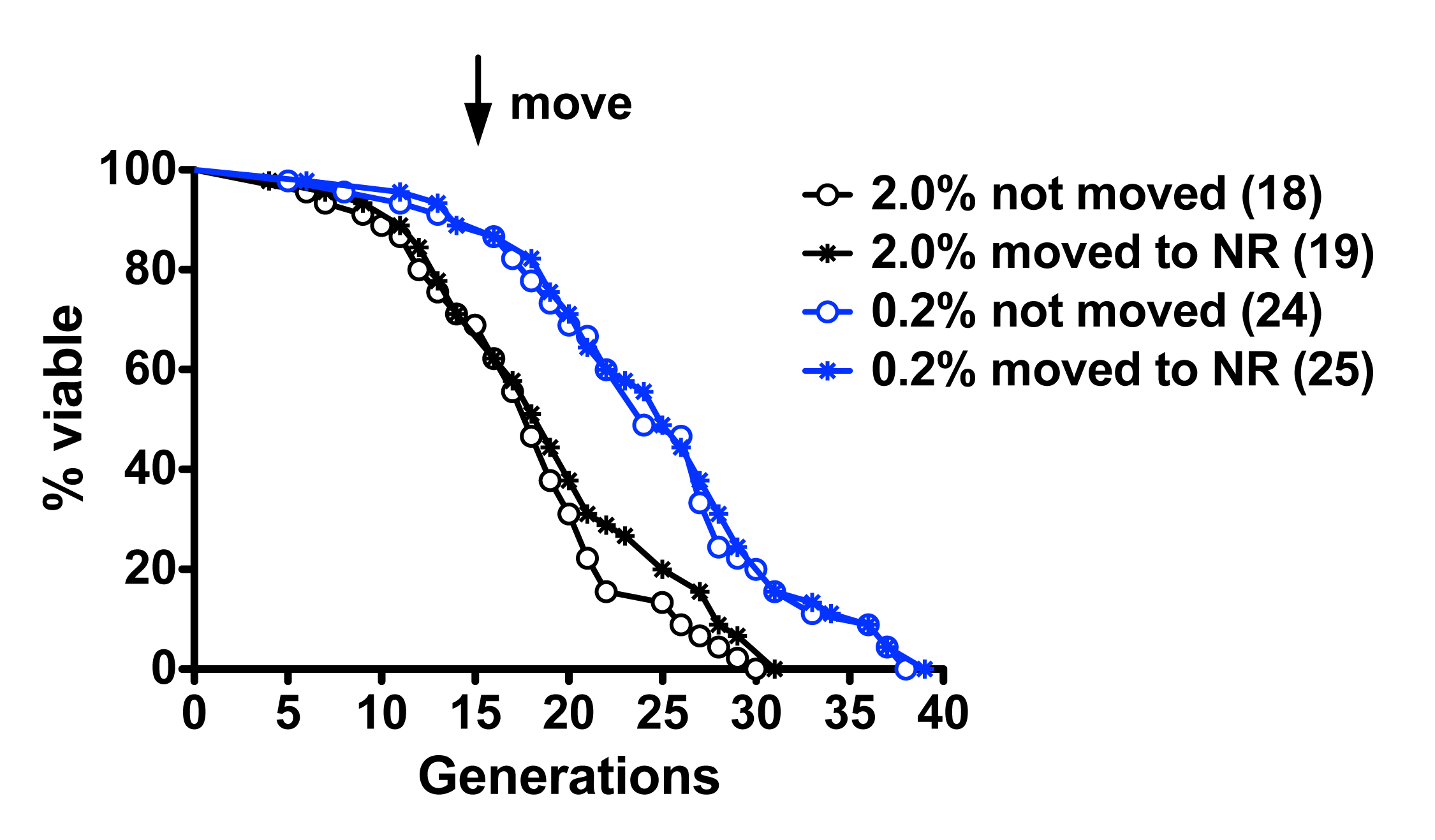

Supplement: S2 Fig — RLS analysis for wild-type BY4741 in 2% glucose and glucose-restricted conditions indicates that supplementation with NR is sufficient to restore the longevity benefit of CR upon migration to new plate locations. n = 45 for each condition. (TIF) [file pbio.1002048.s003.tif]

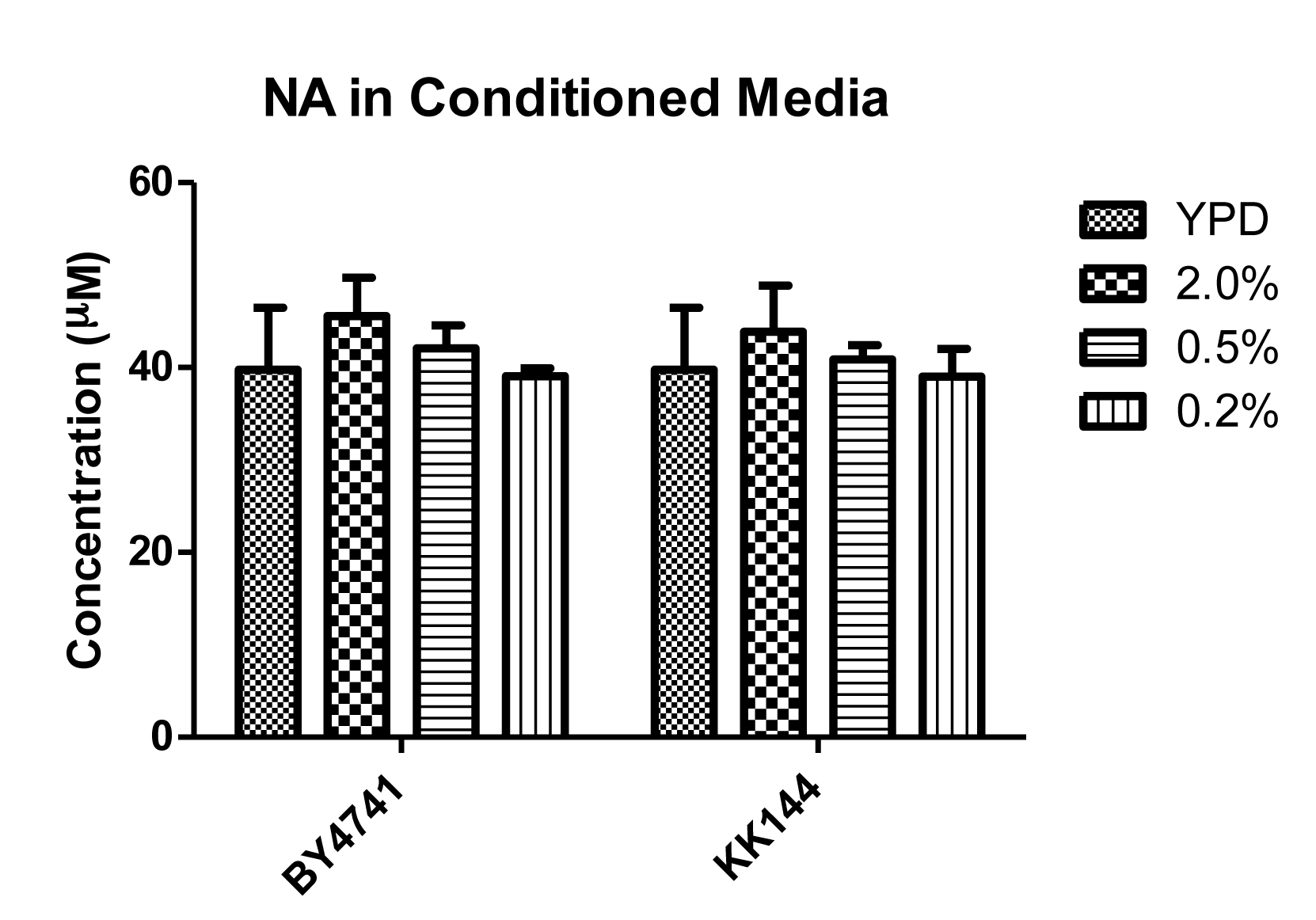

Supplement: S3 Fig — BY4741 and KK144 yeast strains were cultured from OD600 nm 0.0005 to 0.5 in YP media with 2%, 0.5%, or 0.2% glucose. NA concentration from these media and nonconditioned YP were determined by LC-MS [13] with pure NA as standard. (TIF) [file pbio.1002048.s004.tif]

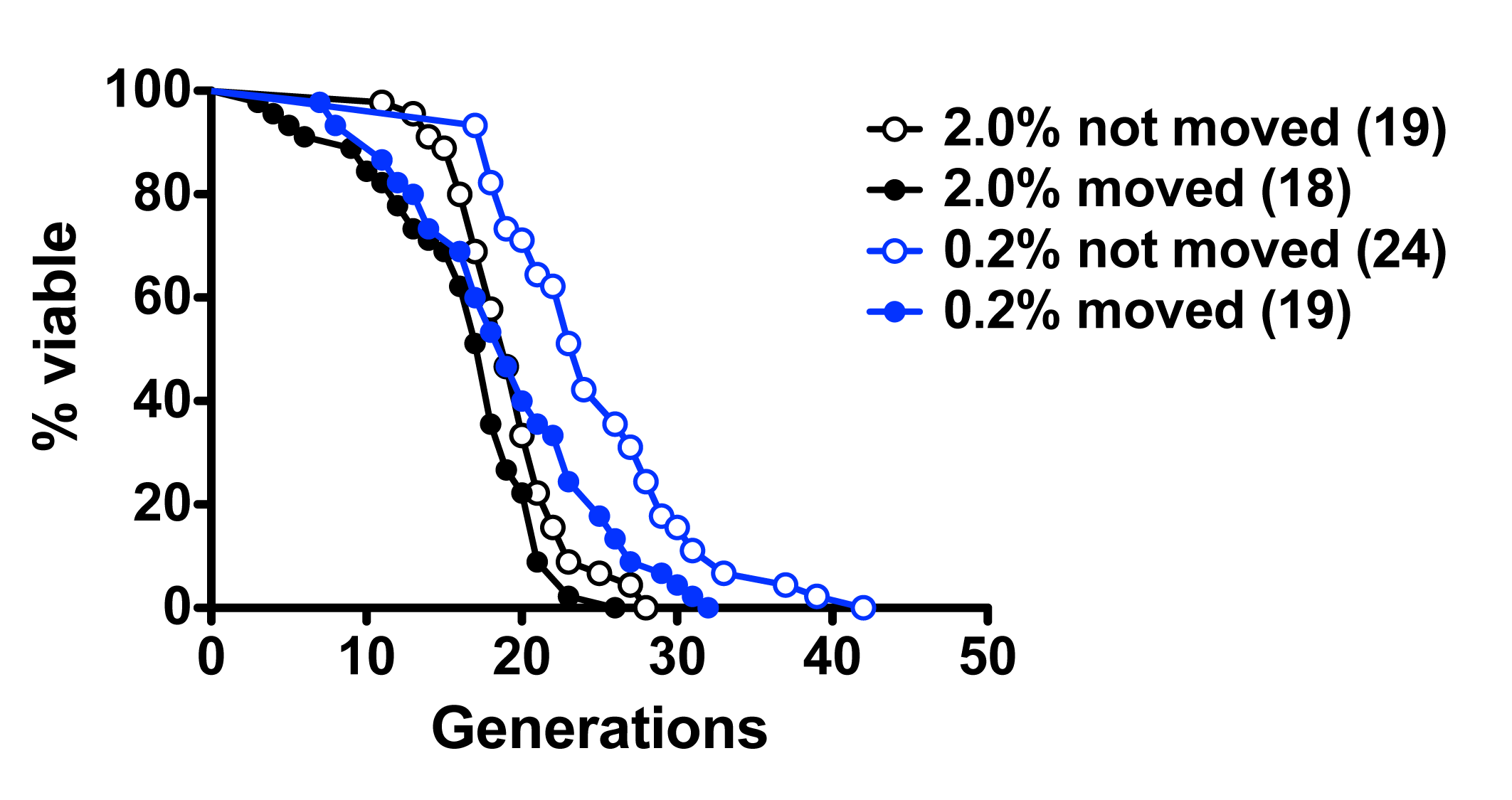

Supplement: S4 Fig — RLS analysis for wild-type BY4741 in 2% glucose and glucose-restricted conditions indicates that keeping yeast mother cells in an always-fresh environment by moving at each generation does not extend lifespan of cells grown at 2% glucose and negates the longevity benefit to glucose-restricted cells. n = 45 for each condition. (TIF) [file pbio.1002048.s005.tif]

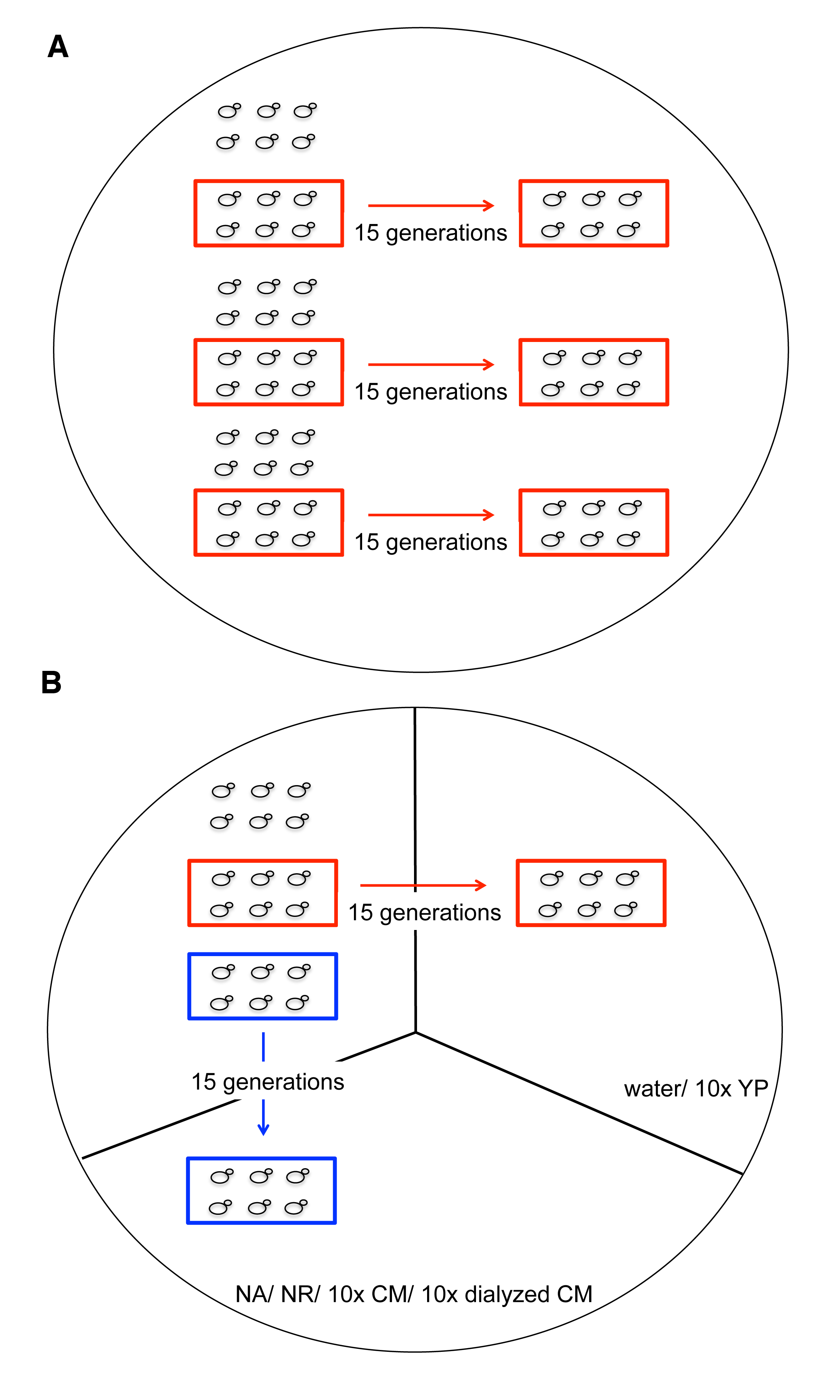

Supplement: S5 Fig — (A) New yeast mother cells were grouped and assigned to be moved or not at the beginning of each RLS experiment. After reaching 15 generations, mother cells assigned to be moved were moved to new locations on the same plate. (B) New yeast mother cells were arrayed on a sector of a plate without supplementation, grouped and assigned to be moved to sectors on the same plate with indicated supplementation or not to be moved. Mother cells to be moved were moved after 15 generations. Water was a control for NA and NR. 10× YP was a control for non-dialyzed or dialyzed conditioned media samples. (TIF) [file pbio.1002048.s006.tif]
